# The Interaction of lncRNA-HEIH and lncRNA-HULC with HBXIP in Hepatitis B Patients

**DOI:** 10.1155/2018/9187316

**Published:** 2018-12-04

**Authors:** Lingjuan Ruan, Lifei Huang, Lilai Zhao, Qiang Wang, Xiaocheng Pan, Anmin Zhang, Qiuping Bai, Zongjun Lv

**Affiliations:** ^1^Department of Laboratory, People's Hospital of Anji, Huzhou, Zhejiang 313300, China; ^2^Department of Galactophore and Thyroid, People's Hospital of Anji, Huzhou, Zhejiang 313300, China; ^3^Department of Orthopaedics and Traumatology, People's Hospital of Anji, Huzhou, Zhejiang 313300, China; ^4^Department of Laboratory, Zhejiang Hospital, Hangzhou 310013, China; ^5^Department of Laboratory, Second People's Hospital of Anji, Huzhou, Zhejiang 313306, China; ^6^Operating Room, People's Hospital of Anji, Huzhou, Zhejiang 313300, China

## Abstract

Hepatitis B virus (HBV) infection is a major risk factor for the development of hepatic cirrhosis (HC) and hepatocellular carcinoma (HCC), which are associated with very high morbidity and mortality rates worldwide. Many studies have shown that long noncoding RNAs (lncRNAs) that are highly expressed in HCC (lncRNA-HEIH) and highly upregulated in liver cancer (lncRNA-HULC) have been implicated in the development and progression of hepatitis B-related HC and HCC. In this study, reverse transcription and quantitative PCR were used to detect the expression of lncRNA-HEIH and lncRNA-HULC and western blot analysis to detect the expression of hepatitis B X-interacting protein (HBXIP). RNA immunoprecipitation was used to detect the interaction of HBXIP with lncRNA-HULC and lncRNA-HEIH. The results showed that lncRNA-HEIH, lncRNA-HULC, and HBXIP were upregulated in hepatitis B patients, particularly those with hepatitis B-related HCC. Both lncRNA-HEIH and lncRNA-HULC interacted with HBXIP. These results suggest that lncRNA-HEIH and lncRNA-HULC interact with HBXIP in hepatitis B-related diseases.

## 1. Introduction

Hepatitis B virus (HBV) infection remains a major public health concern, affecting more than 350 million people worldwide, despite the advent of effective vaccines and other control measures [1–3]. Chronic HBV infection is characterized by the detection of serum hepatitis B surface antigen (HBsAg) after 6 months of infection with continuous liver inflammation and activation of fibrogenic processes, which can lead to hepatic cirrhosis (HC), decompensated (symptomatic) liver disease, and the development of hepatocellular carcinoma (HCC) in 25%–40% of HBV carriers [3]. Thus, it is urgent to identify new and promising diagnostic markers and therapeutic targets for HBV-related diseases. HBV is a double-stranded DNA virus containing four partially overlapping open reading frames, encoding the C, S, and X proteins, and a viral DNA polymerase [4]. Among these four proteins, only the X protein (HBX) has been clearly associated with tumorigenesis [5]. The hepatitis B X-interacting protein (HBXIP) was originally identified by its interaction with the C-terminus of the HBX, which has been found to enhance the growth of hepatoma cells and promote tumorigenesis [6].

Long noncoding RNAs (lncRNAs), a class of RNA segments >200 nucleotides in length, have been considered to be the “noise” of genome transcription owing to their limited protein-coding capacity [7]. With the continued development of viral research, it has been revealed that lncRNAs play important roles in the regulation of protein coding genes, stem cell differentiation, allelic expression, cell cycle control, and cell death [8–11]. Moreover, the aberrant expression of lncRNAs has been widely associated with the physiological and pathological processes of many diseases. Therefore, the potential of lncRNAs as therapeutic targets has been raised and tested in several recent studies. A 16 kb lncRNA found at chromosomal location 6p24.3, which is highly upregulated in liver cancer (lncRNA-HULC) and HCC, is composed of one intron and two exons [12, 13]. In addition, a lncRNA highly expressed in HCC (lncRNA-HEIH) has been associated with disease recurrence and, thus, has been investigated as an independent prognostic factor for overall survival of patients with hepatitis B-related HCC [11]. Although the expression levels of lncRNA-HULC and lncRNA-HEIH were found to be increased in HCC, the underlying molecular mechanisms remain unclear.

The aims of this study were to quantify the expression levels of lncRNA-HULC, lncRNA-HEIH, and HBXIP in patients with HBV infection and hepatitis B-related diseases.

## 2. Materials and Methods

### 2.1. Ethics Statement

The study protocol was approved by the Ethics Committee of People's Hospital of Anji and conducted in accordance with the tenets of the Declaration of Helsinki and the ethical guidelines for medical and health research involving human subjects as established by the National Institutes of Health and the Committee on Human Research of People's Hospital of Anji. Written informed consent was obtained from all patients or their lineal relatives for the use of peripheral blood and liver tissues.

### 2.2. Peripheral Blood Samples

Peripheral blood samples were collected from 75 HBV-positive patients and 25 HBV-negative normal controls who received treatment or medical examinations at People's Hospital of Anji. Patients with infections of HBV or any other virus (e.g., hepatitis virus A, C, D, E, and I) or liver diseases (e.g., hepatic cyst and hepatic metastasis) were excluded from analysis. The 100 study participants were allotted to one of the four following groups (*n* = 25 each): a HBV-positive group (14 females and 11 males with no HBV-related disease); a HBV + HC group (12 females and 13 males with HBV-related disease and HC); a HBV + HCC group (10 females and 15 males with HBV-related disease and HCC); or a control group (13 female and 12 male age-matched controls who were HBV negative with no history of an infectious, psychiatric, neurological, or metabolic disease). The clinical features of all study participants are summarized in Table 1.

### 2.3. Liver Tissues

HBV-positive HCC tissues and corresponding adjacent noncancerous liver tissues (NT) were obtained from patients in the HBV + HCC, HBV-HCC, HBV + NT, and HBV-NT groups who underwent resection in our hospital. On account of the ethical limitation, liver tissues were not collected from the normal controls, HBV carriers, or HBV-positive patients with HC. Thus, normal and cirrhotic liver tissues were not assessed in this study. The clinicopathological characteristics of the study participants are summarized in Table 2.

### 2.4. RT-qPCR

RT-qPCR was used to detect the expression of lncRNA-HULC and lncRNA-HEIH. Total RNA was isolated from the peripheral blood samples, liver tissues, and HepG2.2.15 cells using TRIzol reagent (Invitrogen Corporation, Carlsbad, CA, USA) and then reverse transcribed into cDNA with the Veriti 96-Well Thermal Cycler (Applied Biosystems, Carlsbad, CA, USA) using the PrimeScript RT reagent Kit with gDNA Eraser (TaKaRa Biotechnology (Dalian) Co. Ltd., Dalian, China). Each RT-qPCR reaction contained 2 *μ*L of cDNA, 0.4 *μ*L of forward primer, 0.4 *μ*L of reverse primer, 7.2 *μ*L of H_2_O_2_, and 10 *μ*L SYBR® Green Master MixI (TaKaRa Biotechnology (Dalian) Co. Ltd.). The RT-qPCR protocol consisted of an initial denaturation step at 95°C for 30 s, followed by 40 cycles of amplification at 95°C for 5 s and at 60°C for 34 s, and a final elongation step at 95°C for 15 s. Melting curve analyses were also performed (65.0 to 95.0°C at 0.5°C increments for 5 s). Experimental cycle threshold values were normalized to glyceraldehyde-3-phosphate dehydrogenase (GAPDH) and the relative gene expression levels were determined using the 2^-△△CT^ method [14]. The following primer sequences were used for RT-qPCR: LncRNA-HEIH: forward (F)/reverse (R) CCT CTT GTG CCC CTT TCT T/ATG GCT TCT CGC ATC CTA T; LncRNA-HULC: (F/R) AAC CTC CAG AAC TGT GAT/CAT AAT TCA GGG AGA AAG; 3.5 kb mRNA: (F/R) GCC TTA GAG TCT CCT GAG CA/GAG GGA GTT CTT CTT CTA GG; and GAPDH: (F/R) GAA GGT GAA GGT CGG AGT C/GAA GAT GGT GAT GGG ATT TC.

### 2.5. RNA Immunoprecipitation (RIP)

RIP analyses were performed to detect the interaction between LncRNA-HEIH/LncRNA-HULC and HBXIP using the Magna RIP RNA-Binding Protein Immunoprecipitation Kit (EMD Millipore Corporation, Billerica, MA, USA) in accordance with the manufacturer's instructions. Briefly, liver tissues or harvested cells were lysed in RIP lysis buffer and then incubated with RIP buffer containing magnetic beads conjugated to antibodies (Abs) against HBXIP (Abcam, Cambridge, UK) or normal rabbit immunoglobulin (Ig) G (Abcam). The samples were incubated with proteinase K to digest the proteins, then the coprecipitated RNA was detected by RT-qPCR.

### 2.6. Western Blot Analysis

Total protein was extracted from the liver tissues and HepG2.2.15 cells, then quantified, diluted with 5× loading buffer to the same concentration, denatured at 95°C, separated by sodium dodecyl sulfate-polyacrylamide gel electrophoresis, and then transferred onto polyvinylidene fluoride membranes, which were blocked with 5% skimmed milk at room temperature for 2 h and then incubated with Abs against HBXIP (dilution, 1 : 1000; Abcam) and GAPDH (dilution, 1 : 3000; Abcam) overnight at 4°C. Afterward, the membranes were washed three times with Tris-buffered saline with Tween 20, incubated with horseradish peroxidase-conjugated goat anti-rabbit Ab (dilution, 1 : 4000; Abcam), and washed three times with Tris-buffered saline with Tween 20. Then, the protein bands were visualized using an enhanced chemiluminescence reagent (Santa Cruz Biotechnology Inc., Dallas, TX, USA) and quantified by densitometric scanning with the Fusion-FX7 system (Vilber Lourmat, Collégien, France). The mean optical density of the samples was normalized to that of GAPDH.

### 2.7. Statistical Analysis

All data are expressed as the mean ± standard deviation and were analyzed using the *t*-test, chi-squared test, or one-way analysis of variance with SPSS version 11.5 software (SPSS Inc., Chicago, IL, USA) to determine the significance. A probability (*p*) value of <0.05 was considered statistically significant.

## 3. Results

### 3.1. Expression Levels of LncRNA-HEIH and LncRNA-HULC in Peripheral Blood and Liver Tissues

**Figure 1:**
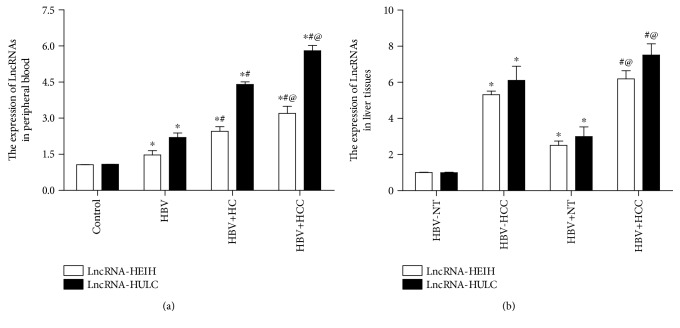
Expression patterns of LncRNA-HEIH and LncRNA-HULC in peripheral blood and liver tissues. (a) Expression levels of LncRNA-HEIH and LncRNA-HULC in peripheral blood (*n* = 25 per group). ^∗^*p* < 0.05 vs. the control group; ^#^*p* < 0.05 vs. the HBV group; @*p* < 0.05 vs. the HBV + HC group. (b) The expression levels of LncRNA-HEIH and LncRNA-HULC in liver tissues (*n* = 25 per group). ^∗^*p* < 0.05 vs. the HBV-NT group; ^#^*p* < 0.05 vs. the HBV + NT group; @*p* < 0.05 vs. the HBV-HCC group.

According to the RT-qPCR results, the expression levels of LncRNA-HEIH and LncRNA-HULC were significantly upregulated in the peripheral blood of HBV-positive patients (HBV, HBV + HC, and HBV + HCC groups), as compared to the control group (*p* < 0.05, Figure 1(a)). Moreover, this increase was more prominent in the HBV + HCC group than the HBV and HBV + HC groups (*p* < 0.05, Figure 1(a)). Similarly, the expression levels of LncRNA-HEIH and LncRNA-HULC were notably increased in the liver tissues of patients with HCC (HBV + HCC and HBV-HCC groups) as compared to corresponding adjacent noncancerous liver tissues (HBV + NT and HBV-NT groups) (*p* < 0.05, Figure 1(b)). In addition, the expression levels of LncRNA-HEIH and LncRNA-HULC were greater in the liver tissues of the HBV + HCC and HBV + NT groups than that in the HBV-HCC and HBV-NT groups, indicating that expression was greater in HBV-positive than HBV-negative liver tissues (*p* < 0.05, Figure 1(b)).

### 3.2. LncRNA-HEIH and LncRNA-HULC Coimmunoprecipitates with HBXIP

**Figure 2:**
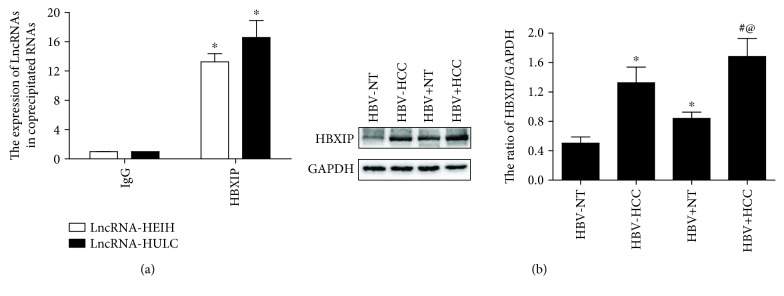
LncRNA-HEIH and LncRNA-HULC coimmunoprecipitates with HBXIP. (a) The expression levels of LncRNA-HEIH and LncRNA-HULC in the samples co-precipitated by HBXIP (*n* = 25 per group). ^∗^*p* < 0.05 vs. the IgG group. (b) The expression levels of HBXIP in liver tissues (*n* = 25 per group). ^∗^*p* < 0.05 vs. the HBV-NT group; ^#^*p* < 0.05 vs. the HBV + NT group; @*p* < 0.05 vs. the HBV-HCC group.

RIP assays were performed to determine whether HBXIP interacts with LncRNA-HEIH and LncRNA-HULC. RNA obtained from the RIP assay using Abs against HBXIP and IgG was subjected to RT-qPCR analysis. The results indicated that the expression levels of LncRNA-HEIH and LncRNA-HULC were greater in the samples pretreated with HBXIP Ab as compared to the those pretreated with IgG (*p* < 0.05, Figure 2(a)), which demonstrated that LncRNA-HEIH and LncRNA-HULC coimmunoprecipitated with HBXIP. Subsequently, the expression levels of HBXIP were quantified in liver tissues by western blot analysis. As shown in Figure 2(b), the expression levels of HBXIP were notably increased in the liver tissues of patients with HCC (HBV + HCC and HBV-HCC groups) as compared to corresponding adjacent noncancerous liver tissues (HBV + NT and HBV-NT groups) (*p* < 0.05) and higher in the HBV + HCC and HBV + NT groups than that in the HBV-HCC and HBV-NT groups (*p* < 0.05).

## 4. Discussion

With the development of high-resolution microarrays and massively parallel sequencing technology, it is widely believed that more than 90% of the human genome is actively transcribed into noncoding RNAs (ncRNAs) and less than 2% actually encodes proteins [15]. The ncRNAs are classified as small ncRNAs (<200 nucleotides) and lncRNAs (>200 nucleotides) according to size. Although these RNAs have been considered to be the “noise” of genome transcription and do not actually participate in gene encoding and protein synthesis directly, they may play significant regulatory roles in a great variety of illnesses, such as hepatitis B infection [16–19]. For example, the small ncRNA miR-137 promotes the expression of HBV genes and viral replication by targeting the expression of the protein inhibitor STAT 2 [20]. Additionally, the miR-99 family promotes HBV replication posttranscriptionally through IGF-1R/PI3K/Akt/mTOR/ULK1 signaling-induced autophagy [21]. In recent years, it has become increasingly obvious that lncRNAs also play critical roles in hepatitis B-related diseases [22, 23]. However, few studies have investigated the mechanisms underlying the involvement of lncRNAs in hepatitis B-related diseases, thus the underlying mechanisms remain unclear. Nonetheless, some previous studies have revealed that lncRNAs play important roles in the regulation of protein coding genes, stem cell differentiation, allelic expression, cell cycle control, and cell death [8–11]. Moreover, lncRNAs are widely involved in physiological and pathological processes, thus lncRNAs have become a main focus of research in the field of molecular biology [24].

The expression levels of lncRNA-HULC and lncRNA-HEIH are relatively high in HCC. LncRNA-HULC is located on chromosome 6p24.3 and regarded as the first lncRNA with highly specific upregulation in HCC, suggesting that it can be used as a promising noninvasive novel biomarker for the diagnosis and/or prognosis of HCC [11, 12]. Du et al. suggested that HBX could promote the expression of lncRNA-HULC in human immortalized normal liver L-O2 cells and hepatoma HepG2 cells, resulting in increased proliferation of hepatoma cells via the suppression of p18 [1, 25]. In addition, Liu et al. demonstrated that a mutation to lncRNA-HULC might contribute to decreased HCC susceptibility in persistent HBV carriers [26]. Moreover, expression of lncRNA-HULC has been positively correlated with the progression of hepatitis B infection and the occurrence of hepatitis B-related diseases, such as HC and HCC. Likewise, in the present study, gradually increased expression of lncRNA-HULC was detected in the peripheral blood samples of the control, HBV, HBV + HC, and HBV + HCC groups. Moreover, lncRNA-HULC expression was greater in the liver tissues of the HBV-positive and HBV-negative HCC groups, as compared to corresponding adjacent noncancerous liver tissues (HBV + NT and HBV-NT groups), and expression of lncRNA-HEIH and LncRNA-HULC was greater in the liver tissues of the HBV + HCC and HBV + NT groups than in the HBV-HCC and HBV-NT groups, which is consistent with the results of a previous study [27]. Similarly, lncRNA-HEIH, another highly expressed lncRNA in HCC, showed identical expression trends with lncRNA-HULC in peripheral blood and liver tissues of patient with hepatitis B or hepatitis B-related diseases. Yang et al. found that lncRNA-HEIH inhibited cell differentiation in the G0/G1 phase and accelerated the carcinogenesis of hepatitis B-related HCC [11, 28]. These results suggest that lncRNA-HULC and lncRNA-HEIH are positively correlated with hepatitis B infection and hepatitis B-related diseases. As we know, lncRNAs are involved in all aspects of gene regulation, including chromosome dosage compensation, imprinting, epigenetic regulation, nuclear and cytoplasmic trafficking, and transcription, as well as mRNA splicing and translation [29]. In the present study, the positive interactions of HBXIP with lncRNA-HULC and lncRNA-HEIH were further investigated by RIP analysis, which showed that HBXIP expression was greater in HBV-positive than HBV-negative HCC. Wang et al. indicated that HBXIP might play an important role in tumorigenesis by enhancing angiogenesis in HCC [6]. Therefore, it is possible that high expression of lncRNA-HULC and lncRNA-HEIH promotes the expression of HBXIP, which results in increased HBV replication and the occurrence of hepatitis B-related diseases.

In summary, the present findings demonstrate that the interactions of lncRNA-HULC and lncRNA-HEIH with HBXIP might be involved in the occurrence of hepatitis B-related diseases. Understanding the relationship of lncRNA-HULC and lncRNA-HEIH with HBXIP in hepatitis B and hepatitis B-related diseases may lead to the development of novel therapeutic interventions to ameliorate hepatitis network dysfunction and associated morbidities.

## Figures and Tables

**Table 1:** Clinical features of patients.

Parameter	Control	HBV	HBV + HC	HBV + HCC	*p*
Gender					
Female	13	14	12	10	>0.05
Male	12	11	13	15	
HBsAg					
Negative	25	0	0	0	<0.05
Positive	0	25	25	25	
Age	45.07 ± 15.06	40.18 ± 16.25	42.31 ± 8.56	45.62 ± 11.28	>0.05
ALP (U/L)	55.58 ± 12.01	91.46 ± 12.96	121.77 ± 16.89	157.29 ± 21.78	<0.05
ALB (g/L)	48.44 ± 8.88	43.32 ± 7.58	28.21 ± 6.69	30.37 ± 6.84	<0.05
TBIL (*μ*mol/L)	15.23 ± 3.67	22.24 ± 4.52	33.52 ± 7.17	40.44 ± 8.03	<0.05
DBIL (*μ*mol/L)	4.28 ± 1.13	8.44 ± 1.61	17.22 ± 3.35	27.24 ± 5.98	<0.05
AFP (*μ*g/L)	4.95 ± 1.13	26.83 ± 4.67	52.79 ± 7.76	122.74 ± 18.76	<0.05
ALT (U/L)	25.48 ± 6.68	181.67 ± 21.90	69.28 ± 11.11	78.24 ± 10.94	<0.05
AST (U/L)	22.14 ± 5.11	196.75 ± 20.29	89.89 ± 13.22	102.25 ± 14.11	<0.05
HBV-DNA (copies/mL)	0	(7.12 ± 0.28) × 105	(2.23 ± 0.47) × 104	(7.35 ± 0.55) × 104	<0.05

HBsAg: hepatitis B surface antigen; ALP: alkaline phosphatase; ALB: albumin; TBIl: total bilirubin; DBIl: direct bilirubin; AFP: alpha-fetoprotein; ALT: alanine aminotransferase; AST: aspartate aminotransferase.

**Table 2:** Clinical features of patients.

Parameter	HBV + HCC	HBV-HCC	*p*
Gender			
Female	10	12	>0.05
Male	15	13	
HBsAg			
Negative	0	25	<0.05
Positive	25	0	
Age	45.62 ± 11.28	44.71 ± 9.98	>0.05
ALP (U/L)	166.29 ± 36.12	150.49 ± 29.98	>0.05
ALB (g/L)	34.56 ± 10.18	33.33 ± 17.24	>0.05
TBIL (*μ*mol/L)	42.49 ± 10.36	45.05 ± 6.21	>0.05
DBIL (*μ*mol/L)	31.75 ± 4.98	30.23 ± 5.69	>0.05
AFP (*μ*g/L)	125.78 ± 19.27	126.09 ± 22.08	>0.05
ALT (U/L)	90.57 ± 12.14	91.07 ± 25.07	>0.05
AST (U/L)	111.15 ± 9.72	126.77 ± 25.69	>0.05
HBV-DNA (copies/mL)	(8.21 ± 0.69) × 10^4^	0	<0.05

HBsAg: hepatitis B surface antigen; ALP: alkaline phosphatase; ALB: albumin; TBIl: total bilirubin; DBIl: direct bilirubin; AFP: alpha-fetoprotein; ALT: alanine aminotransferase; AST: aspartate aminotransferase.

## Data Availability

The data used to support the findings of this study are available from the corresponding author upon request.

## References

[1] Wen W. H., Huang C. W., Chie W. C. (2016). Quantitative maternal hepatitis B surface antigen predicts maternally transmitted hepatitis B virus infection. *Hepatology*.

[2] Wen W. H., Lai M. W., Chang M. H. (2015). A review of strategies to prevent mother-to-infant transmission of hepatitis B virus infection. *Expert Review of Gastroenterology & Hepatology*.

[3] Yuen M.-F., Chen D.-S., Dusheiko G. M. (2018). Hepatitis B virus infection. *Nature Reviews Disease Primers*.

[4] Birrer R. B., Birrer D., Klavins J. V. (2003). Hepatocellular carcinoma and hepatitis virus. *Annals of Clinical and Laboratory Science*.

[5] Fujii R., Zhu C., Wen Y. (2006). HBXIP, cellular target of hepatitis B virus oncoprotein, is a regulator of centrosome dynamics and cytokinesis. *Cancer Research*.

[6] Wang F., Fei H., Qi B., Yao S., Chang Z. (2012). Overexpression of hepatitis B x-interacting protein in HepG2 cells enhances tumor-induced angiogenesis. *Molecular and Cellular Biochemistry*.

[7] Wilusz J. E., Sunwoo H., Spector D. L. (2009). Long noncoding RNAs: functional surprises from the RNA world. *Genes & Development*.

[8] Jain S., Thakkar N., Chhatai J., Pal Bhadra M., Bhadra U. (2016). Long non-coding RNA: functional agent for disease traits. *RNA Biology*.

[9] Vance K. W., Ponting C. P. (2014). Transcriptional regulatory functions of nuclear long noncoding RNAs. *Trends in Genetics*.

[10] Dinger M. E., Amaral P. P., Mercer T. R. (2008). Long noncoding RNAs in mouse embryonic stem cell pluripotency and differentiation. *Genome Research*.

[11] He Y., Meng X. M., Huang C. (2014). Long noncoding RNAs: novel insights into hepatocelluar carcinoma. *Cancer Letters*.

[12] Panzitt K., Tschernatsch M. M. O., Guelly C. (2007). Characterization of HULC, a novel gene with striking up-regulation in hepatocellular carcinoma, as noncoding RNA. *Gastroenterology*.

[13] Zhang Y., Li Z., Zhang Y., Zhong Q., Chen Q., Zhang L. (2015). Molecular mechanism of HEIH and HULC in the proliferation and invasion of hepatoma cells. *International Journal of Clinical and Experimental Medicine*.

[14] Schmittgen T. D., Livak K. J. (2008). Analyzing real-time PCR data by the comparative C(T) method. *Nature Protocols*.

[15] Esteller M. (2011). Non-coding RNAs in human disease. *Nature Reviews Genetics*.

[16] de Almeida R. A., Fraczek M. G., Parker S., Delneri D., O'Keefe R. T. (2016). Non-coding RNAs and disease: the classical ncRNAs make a comeback. *Biochemical Society Transactions*.

[17] Varela M. A., Roberts T. C., Wood M. J. A. (2013). Epigenetics and ncRNAs in brain function and disease: mechanisms and prospects for therapy. *Neurotherapeutics*.

[18] Yu K., Shi G., Li N. (2015). The function of MicroRNA in hepatitis B virus-related liver diseases: from dim to bright. *Annals of Hepatology*.

[19] Wu S. Y., Lan S. H., Liu H. S. (2016). Autophagy and microRNA in hepatitis B virus-related hepatocellular carcinoma. *World Journal of Gastroenterology*.

[20] Pan Y., Dai J., Liao Y., Yu Q. (2017). MicroRNA-137 promotes hepatitis B virus gene expression and replication via targeting the protein inhibitor of activated STAT 2. *Pharmazie*.

[21] Lin Y., Deng W., Pang J. (2017). The microRNA-99 family modulates hepatitis B virus replication by promoting IGF-1R/PI3K/Akt/mTOR/ULK1 signaling-induced autophagy. *Cellular Microbiology*.

[22] Niu J., Lin Y., Liu P., Yu Y., Su C., Wang X. (2016). Microarray analysis on the lncRNA expression profile in male hepatocelluar carcinoma patients with chronic hepatitis B virus infection. *Oncotarget*.

[23] Qiu L., Wang T., Xu X., Wu Y., Tang Q., Chen K. (2017). Long non-coding RNAs in hepatitis B virus-related hepatocellular carcinoma: regulation, functions, and underlying mechanisms. *International Journal of Molecular Sciences*.

[24] Guil S., Esteller M. (2015). RNA-RNA interactions in gene regulation: the coding and noncoding players. *Trends in Biochemical Sciences*.

[25] Du Y., Kong G., You X. (2012). Elevation of highly up-regulated in liver cancer (HULC) by hepatitis B virus X protein promotes hepatoma cell proliferation via down-regulating p18. *The Journal of Biological Chemistry*.

[26] Liu Y., Pan S., Liu L. (2012). A genetic variant in long non-coding RNA HULC contributes to risk of HBV-related hepatocellular carcinoma in a Chinese population. *PLoS One*.

[27] Pan Y. F., Qin T., Feng L., Yu Z. J. (2013). Expression profile of altered long non-coding RNAs in patients with HBV-associated hepatocellular carcinoma. *Journal of Huazhong University of Science and Technology Medical Sciences*.

[28] Yang F., Zhang L., Huo X. S. (2011). Long noncoding RNA high expression in hepatocellular carcinoma facilitates tumor growth through enhancer of zeste homolog 2 in humans. *Hepatology*.

[29] Clark M. B., Mattick J. S. (2011). Long noncoding RNAs in cell biology. *Seminars in Cell & Developmental Biology*.

